# The Social Determinants of Adverse Childhood Experiences: An Intersectional Analysis of Place, Access to Resources, and Compounding Effects

**DOI:** 10.3390/ijerph191710670

**Published:** 2022-08-29

**Authors:** Sayil Camacho, Sarah C. Henderson

**Affiliations:** 1 Equity Research Cooperative, 2929 Arch Street Suite 1700-#3207, Philadelphia, PA 19104, USA; 2 Department of Health Policy, Vanderbilt University School of Medicine, 1211 Medical Center Drive, Nashville, TN 37232, USA; sarahclarkhenderson@alumni.emory.edu

**Keywords:** adverse childhood experiences, child abuse, child neglect, household dysfunction, intersectionality

## Abstract

Children across all races/ethnicities and income levels experience adverse childhood experiences (ACEs); however, historically excluded children and families must contend with added adversities across ecological levels and within higher-risk conditions due to systemic inequality. In this grounded theory study, the authors examined how health and social service providers (*N* = 81) from rural and urban counties in Tennessee provided services to low-income families, children exposed to opioids, and children of immigrants. Guided by an intersectional framework, the authors examined how rural and urban settings shaped higher risk conditions for ACEs and impeded access to resources at the individual, group, and community levels. Findings from this study identified additionally marginalized subpopulations and demonstrated how inequitable environments intersect and compound the effects of ACEs. The authors present their *Intersectional Nature of ACEs Framework* to showcase the relationship between high-risk conditions and sociopolitical and economic circumstances that can worsen the effects of ACEs. Ultimately, the *Intersectional Nature of Aces Framework* differentiates between ACEs that are consequences of social inequities and ACEs that are inflicted directly by a person. This framework better equips ACEs scholars, policymakers, and stakeholders to address the root causes of inequality and mitigate the effects of ACEs among historically excluded populations.

## 1. Introduction

Child abuse, neglect, and household dysfunction are collectively referred to as *adverse childhood experiences* (ACEs) and are associated with worse outcomes over a child’s lifetime [1]. It is important to establish that although ACEs are not determined by a child’s race, class, or gender, they are more prevalent among historically excluded populations in communities made vulnerable by poverty and scarce public resources [2,3]. Previous research findings have suggested the significant impacts of systemic inequality, as historically marginalized children, families, and communities are more likely to live in high-risk environments that compound ACEs [4,5].

While ACEs scholarship has generally emphasized the 10 traditional adverse childhood experiences, emerging studies have acknowledged the importance of ACEs related to environmental factors that may disproportionately affect marginalized children and families [2,6]. Subsequent studies have identified expanded ACEs related to environmental factors (i.e., neighborhood violence, homelessness, foster care, bullying, and racism); this research has: (1) advanced dialogue around the diversity of ACEs; (2) examined the relationship between ACEs and child demographics; and (3) demonstrated which populations of children are more likely to experience ACEs [2,7,8]. However, scholars have not critically examined how systemic inequality shapes lived realities to understand the relationship among high-risk environments, access to resources, and ACEs. In addition, researchers have not explored how intersectional experiences within high-risk environments may compound the effects of ACEs and additionally marginalize populations within and across ecological levels (i.e., individual, group, or community units of analysis). 

To account for children’s diverse experiences of abuse, neglect, and household dysfunction at the individual, group, and community levels, we examined urban and rural environments in which ACEs occur. We utilized 81 in-depth interviews with health and social service providers in the state of Tennessee to understand how historically excluded populations—that is, low-income families, children exposed to opioids, and children of immigrants—access resources and experience place-based challenges that raise high-risk conditions. Interview participants were at the frontlines of mitigating ACEs, playing a key role in helping families access vital resources and services [9]. Guided by a process-centered intersectionality framework [10,11,12], our grounded theory research design assumed there are various social, political, and economic inequities that perpetuate conditions of oppression among historically excluded children, families, and communities, which permitted a critical understanding of underlying issues that create high-risk environments [10,13,14]. 

We present the *Intersectional Nature of ACEs Framework* to showcase how environments shape high-risk conditions; link intersectional experiences of recognized and unrecognized individuals, groups, and populations; and have confounding effects related to ACEs. While quantitative, population-level studies can describe the existence of an ACE or multiple ACEs, our study identifies the underlying issues that construct high-risk environments and worsen ACEs for children, families, and communities. 

### 1.1. Background

We utilized the concept of intersectionality to guide our review of the ACEs literature and identify the extent to which empirical studies have included systemic inequality across ecological levels. Intersectionality is a theoretical framework embedded in research studies that seek to support a nuanced understanding of how various forms of experienced inequality interface with one another and exacerbate marginalization among historically excluded populations. This positionality challenges single-axis frameworks and supports the ability to understand within-group differences at the individual (micro), group (mezzo), or community (macro) level [15,16,17]. In reviewing the ACEs literature, we sought to understand how previous studies have operationalized intersectionality and the extent to which findings have considered additionally marginalized subpopulations within and across ecological levels. Our literature review identifies 20 studies that espoused an intersectional framework directly or indirectly to examine the ACEs phenomena among historically excluded populations. 

### 1.2. Expanded ACEs and Historically Excluded Populations

To date, ACEs scholarship has identified the following experiences as forms of expanded ACEs: neighborhood violence, witnessing violence, bullying, poverty, homelessness, and foster care [8,18,19,20,21,22,23]. Although studies investigating expanded ACEs have focused on how children interface with environments, most have not described upstream factors that raise risk for ACEs and contribute to experiences of co-occurring ACEs among children who experience the consequences of systemic inequality. For example, two studies that identified expanded ACEs utilized the differential exposure hypothesis to contextualize the examination of which groups or populations were more likely to be exposed to ACEs per gender, economic status, and/or race [6,24]. Recent studies have also linked newly identified ACEs with higher risks for negative outcomes, such as poverty, poor mental health, behavior problems, and risky health behaviors [7,8,18,21,22,25]. 

These and other studies have confirmed that historically excluded populations (e.g., Black, Indigenous, People of Color also referred to as people of the global majority) experience additional challenges and are more likely to experience ACEs as a result of systemic inequality and how it shapes their identities and lived realities [5,24,26,27,28,29]. While we acknowledge the importance of identifying which populations of children are at risk, we argue that it is critical to establish how systemic inequality within and across ecological levels shapes high-risk environments. 

### 1.3. Systemic Inequality and Co-Occurring ACEs

Although the aforementioned studies built upon ACEs phenomena among historically excluded populations, they generally did not establish how these experiences are constructed by political and socioeconomic systems contributing to high-risk environments. Moreover, the super-majority of studies did not examine how political and socioeconomic factors contribute to experienced adversity among children. In fact, we only identified one study that considered the nuance of sociocultural factors that shaped high-risk environments associated with ACEs [5]. While it is helpful to know which populations need additional support to address ACEs and build resilience among children, it is even more important to know *why* higher risk conditions exist and to address root causes of inequities that increase the risk of ACEs.

ACEs scholars refer to the differential burden of ACEs as a *co-occurring phenomenon*, and this, too, is experienced at higher rates among historically marginalized populations [25,29]. The differential burden concept explains why certain groups may experience worse outcomes from ACEs linked to demographic characteristics or social identities [27,29]. Limited access to resources based on one’s identity may also play a role in which children affected by ACEs are most likely to access treatment and support. The ability of stakeholders, clinicians, and policymakers to distinguish between demographics and the inequitable environments that raise high-risk conditions for communities made vulnerable is critical to mitigating deficit perspectives and facilitating comprehensive support for children who experience ACEs. For example, higher exposure to ACEs should not be linked to the status of *being* part of the global majority or belonging to a historically marginalized population; rather, if ACEs are a universal experience, high-risk conditions must be regarded as imposed-upon environments that compound ACEs and inflict additional harm on historically excluded children, families, and communities. 

### 1.4. Contributions to the Literature 

In our review of the literature, no study operationalized a process-centered intersectionality framework or fully discussed how an intersectional approach could advance the analysis of ACEs. The two studies that examined the intersection of ACEs and demographic factors did not expand upon how policies and systems raise the risk of ACEs and stigmatize populations experiencing a higher burden of ACEs; rather, the concept of intersectionality was used to contextualize the introduction of the topic and explain the intersection of factors connected with ACEs [6,30]. Similarly, a study on ACEs and wellness outcomes among Black men who have sex with men introduced the concept of the intersection of identities and dual experiences, but the authors did not consider how researchers might use an intersectional approach to expand upon the understanding of ACEs [29]. 

Our study contributes to the ACEs literature within various dimensions. First, our approach expands upon the differential exposure hypothesis by explaining the conditions historically marginalized populations are more likely to experience and, thereafter, bridges the topics of high-risk conditions and ACEs. Second, we expand upon the differential burden concept by explaining how policies and systems determine access to vital resources and services for children experiencing ACEs; access to resources and services are important in reducing the risk of intergenerational trauma, abuse, and household dysfunction among children and families [2,31]. We also build upon previous ACEs studies that have espoused an intersectional perspective by being the first to operationalize a process-centered intersectional framework. 

### 1.5. The Current Study

The purpose of this grounded theory study was to examine how health and social service providers (*N* = 81) from rural and urban counties in Tennessee provided services to low-income families, children exposed to opioids, and children of immigrants. Specifically, we explored two guiding research questions: (1) How do rural and urban environments shape high-risk conditions for children, families, and communities? (2) How do high-risk conditions compound ACEs and impede access to resources at individual, group, and community levels? 

Camacho and Henderson were researchers for the *Policies for Action Research Hub* study at Vanderbilt University funded by the Robert Wood Johnson Foundation. Camacho was a *Policies for Action Research Hub* postdoctoral fellow and responsible for developing and leading the qualitative data collection and analysis effort. Henderson was a Senior Health Services Research Analyst who contributed to quantitative and qualitative data collection, management, and analysis. This study is part of a larger, transdisciplinary study that seeks to identify policies and practices in the state of Tennessee that can improve the health and education outcomes among the state’s most vulnerable children. The research team constituted nine investigators from the Vanderbilt University School of Medicine’s Department of Health Policy and Peabody College of Education and Human Development. Collectively, researchers utilized quantitative and qualitative methods to understand the complex relationships that exist between state-level policies and access to services that either facilitate or impede the ability for children and their families to receive vital health and education services. 

## 2. Materials and Methods

### 2.1. Recruitment and Sample

The research team constructed a sampling frame to recruit individuals working in state agencies, local health departments, safety-net clinics, schools, and nonprofits. The research team utilized purposive sampling to prioritize organizations that served marginalized families and were situated in different geographic areas that were designated as economically distressed counties, had higher rates of neonatal abstinence syndrome (NAS), and had higher percentages of Latinx/e populations. Thereafter, random sampling was utilized to select participants from each type of organization to ensure diverse organization representation. They purposely selected two counties with the highest incidences of NAS, and the highest rates of Latinx/e children of immigrants.

Administrative data was used to stratify the sample based on region designation (i.e., west, middle, east) by the Tennessee Department of Education and urbanicity (i.e., town, city, suburb, rural). In addition, distances were calculated using the percent of marginalized populations within each county to select closest to the average Mahalanobis score (this measures the distance between two points and the distribution). After finalizing the sampling frame, Camacho contacted interview participants via email and phone to request an interview either in person or over the phone. Each in-depth interview was 60 to 90 min in length, and we attempted to establish a supportive interview environment by acknowledging the significance of providing support to historically marginalized populations. 

We conducted 47 interviews in 26 counties (of the 95 in Tennessee) in all three regions of the state; nine of these counties were designated as economically distressed. Most interviews were in-depth, one-on-one interviews (34) conducted by two or three members of the research team. When possible, we encouraged interview participants to invite work colleagues to be part of the in-depth interview process to better understand organizational policies and practices from various perspectives; subsequently, we conducted a total of 13 focus groups with two to nine interview participants per focus group. Interview participants worked in the following organizations, and we include the number of interviews conducted with each organization: community advocacy organizations (*n* = 5), community anti-drug coalitions (*n* = 8), community mental health centers (*n* = 3), coordinated school health directors (*n* = 15), county health departments (*n* = 4), federally qualified health centers (*n* = 3), Medicaid (*n* = 1), neighborhood health centers (*n* = 3), opioid treatment programs (*n* = 1), school-based health centers (*n* = 2), and Tennessee early interventions systems (*n* = 1). A total of 81 health and social service providers participated in the interviews. The study was approved by the Institutional Review Board at Vanderbilt University, and informed consent was obtained from all interview participants.

### 2.2. Data Analysis 

We employed a grounded theory methodology to examine data corresponding to our research questions and systematically utilized comparative analysis to construct a theory from the dataset [32]. Modeling the intersectional grounded theory research design [33], we assumed a process-centered intersectional approach to guide our understanding of within-group differences (mezzo) and help identify how inequality functions within structured mechanisms. As developed by McCall (2005) and Davis (2008), an intersectional process-centered method does not limit the intersections of experiences to individuals (micro) but suggests that group intersectional analysis (mezzo and macro) reveals how systemic inequality operates [11,12,14]. Therefore, our data analysis process differentiated between personal and collective experiences to better understand how external factors are placed upon children, families, and communities [34]. In line with this approach, we conceptualized three waves of data analysis whereby the first data analysis utilized the interview guide to develop a priori codes, the second used our theoretical framework to identify emergent constructs pertaining to systemic inequality, and the third catalogued systems and processes that prohibited access to resources and services across ecological levels. 

All interviews were transcribed verbatim, and we used NVivo (version 12, produced by QSR International, London, UK), a qualitative software program, to organize the qualitative data and codebook for the qualitative data analysis process. We began the first wave of data analysis by applying Boyatzis’s (1998) categorical analysis and derived a priori themes from the initial interview protocol [35]. The following themes helped frame the analysis: (1) information about the organization and role of the service provider; (2) current public assistance policies and support for vulnerable populations; (3) health and mental health resources for immigrant populations; (4) barriers to service awareness and strategies implemented to address systematic barriers; (5) prenatal and postnatal support for opioid users; (6) neonatal abstinence syndrome and treatment; (7) school-based healthcare resources; and (8) prohibitive policies, systems, and structures. In particular, sections in the interview protocol provided interview participants with the opportunity to respond to the changing needs of historically marginalized families, regardless of the organizational type, so that we could uniformly gauge the capacity of organizations to provide important services and resources during the data analysis. 

Thereafter, deductive codes emerged respective to each category and our intersectional process-centered theoretical framework. This thematic analysis produced additional codes that expanded across the categories, including: (1) the imposed-upon environments that historically marginalized families have to navigate at micro, meso, and macro levels; (2) how inequality perpetuates intersectional marginality, prohibiting access to services and/or compounding ACEs; (3) identified and non-identified subpopulations as a consequence of inequality; (4) the different forms of burdens that communities made vulnerable have to navigate and how inequality perpetuates burdens; and (5) the compounding effects of high-risk conditions related to ACEs. Since process-centered intersectionality emphasizes the examination of recognized and unrecognized populations, our data analysis included identifying subpopulations that are additionally impacted by high-risk environments not commonly accounted for in research studies. These identities extend beyond general demographic information such as race, gender, or family structure. 

As we engaged in the coding process during the first and second waves of the data analysis, we catalogued systems and processes that prohibited access to resources and support across ecological levels and according to place. This process constituted a third wave of data analysis, and we produced a master document that differentiated the types of prohibitive policies, systems, and processes by type of organization and in relationship to lack of monetary resources, administrative burdens, and need for new programs and sources of support. 

Our codebook was piloted three times among Camacho and Henderson, using the same five interviews. Codes were modified until there was 90% agreement among the researchers when coding a sample of responses. Upon establishing intercoder agreement, each interview was coded twice. True to the study design, diverse and conflicting data was regarded as indicative of the complexity of the phenomena. This approach assumed that the findings were not contradictory but, rather, multifaceted. Altogether, the three waves of data analysis were utilized to answer the guiding research questions, and our grounded theory approach ensured that the development of our framework was anchored in the dataset post data analysis.

### 2.3. Limitations

Given the extent to which process-centered intersectionality recognizes and elevates experiential knowledge, the lack of participation from historically excluded populations who cannot access health and social services is a methodological limitation. By focusing on the experiences of health and social service providers, this study elevates the experiences of a more privileged population. Therefore, findings from this are not meant to discount the real and lived experiences of historically marginalized populations. Rather, the experiences of service providers are contextualized within our analysis of power per the critical framework employed. 

## 3. Results

Overall, the interviewees identified several factors that shape high-risk environments and compound exposure to ACEs within their service type and community context. Interviewees also described limited access to resources and support due to policy constraints at the local, state, and federal levels which further compounded negative outcomes among children in high-risk environments. 

### 3.1. Salience of Place: Rural, Urban, and Economic Characteristics

Across Tennessee, health and education service providers spoke in-depth about the *salience of place*—the ways that rural, urban, and economic status of a county bring forth unique challenges that raise high-risk conditions for the populations served. While both urban and rural communities experience different place-based challenges, in Tennessee, the act of living in a rural community poses additional challenges related to limited socioeconomic opportunities (i.e., employment and living wages) and mobility (i.e., distance between resources and lack of public transit). For many of the interviewees, namely the majority of those who served rural communities with higher poverty levels, poverty brought forth other forms of adversity that either (a) shaped higher-risk environments which subsequently increased the risk of experiencing ACEs or (b) presented additional challenges when children and families needed to access resources and support. To illustrate differences between factors that shape higher-risk environments and the ways in which place can prohibit access to resources and support, we first present place-based challenges linked to poverty followed by related ACEs that may result due to higher-risk environments. Referenced factors that shape higher-risk environments included: food deserts, insufficient number of affordable housing programs due to lower population density, lack of public transit infrastructure, limited number of healthcare and hospital services, workforce recruitment and retainment issues for service providers, limited number of translation services for non-English speaking people, and insufficient number of beds at opioid treatment centers. These experienced place-based conditions meant individuals, families and communities were more likely to experience food, housing, and transit insecurity across ecological levels, as well as have unmet mental and physical health needs. The relationship between higher-risk environments and adverse childhood experiences translated into increased risk of experiencing ACE(s), as well as limited access to resources and support. For example, families in rural or sparsely populated counties lacked access to public transit in under resourced towns, which limited their ability to travel to receive health and social services. According to service providers, lack of resources and access to services contributed to intergenerational cycles of poverty, addiction, and other household-level crises that negatively affected children. Consequently, place was an important factor that often contributed to risk of exposure to ACEs, root causes of poverty, and the accessibility of resources and supports for children and families. The significance of place and factors that shape higher-risk environments and limited access to resources are illustrated in the following interview excerpts:

“So, we didn’t have a social worker. And the reason that the social worker is so important is, we are in a rural area. Our poverty rate here in [County] is 43.7%. So, I have a lot of students who live in isolation. We have a lot of students that are in transit all the time. I guess they would technically fit under homelessness because they’re living with someone else, they’re here, there, they’re really hard—you know, those are the kids that are truant. Those are the kids who have health needs. So, today, this afternoon, I’ll be talking with the department of children’s services about continuing that funding for the social worker…. So, this is something that is needed, because our students who are in poverty, as you know, they’re about seven times more likely to have mental issues or be living in a home where someone has mental issues. And that connection between the classroom and that student’s parents, the caregiver, is almost nonexistent. We only have about 60% of the people here who have internet, and then they can’t afford a phone a lot of times, or if they do afford the phone, they can’t keep it on. Yeah, so the social worker has been able to reach out and go to the home, knock on the door, and say, “Hey, I’m from the school, you know, what do you need. How can we help you and how can you help us, you know, to better educate your child?” It has been a wonderful godsend having her to be able to reach out.” (Coordinated school health director in a rural, economically distressed county)

“So, we have 50 kids that are not being seen at least on school-based therapy. We try to find them places outside the school. See, here’s the issue. We need the school-based therapy. And I’m not speaking just for my school. I’m talking about school in general, because (a) there’s a transportation issue, especially in your high-poverty school districts, and (b), if parents have a car, they’re at work, and they don’t have—you know, these low-paying jobs do not offer sick days and, you know, time off and all that kind of stuff. So, parents are—cannot really take off and take the child to therapy, so—at least our Medicaid people are in that boat. And there are others, you know, insurance folks are in the same boat. I mean, we’re seeing insured kids at the school, too, but Medicaid kids are top priority. So that’s—we have got to have more focus on school-based therapy.” (Coordinated school health director in a city-adjacent county)

From the perspective of service providers, the less competitive wages in rural communities negatively affected their respective organization’s ability to hire and/or retain top-qualified health and education service providers in the most high-need, high-risk communities. A rural community may or may not have a hospital or urgent-care services, and schools are often the only regular healthcare service that a child receives, especially when the child does not have a pediatric home; meaning, the child’s family or caretaker has not established a primary physician for the child due to an inaccessible healthcare infrastructure per the nature of rurality and/or the lack of public transit.

“The other thing that is—that we have a need for is more mental health inside the schools, and in an impoverished area like this, nobody wants to come here. We have been through five school-based mental health counselors in the last 3 years. We have a partnership with a local mental health agency, and they cannot keep someone employed inside this school. These people are getting money to go elsewhere and, you know, work in better places for more money. So, you know, it’s really impacting the kids, because we also have a very high suicide rate. For example, you know, our youngest one is 9 years old.” (Coordinated school health director in a rural, economically distressed county).

### 3.2. Salience of Place: Sociopolitical Context and the Culture of Care

Given the limitations imposed by rurality, we found that place also largely influenced how the culture of care was organized. Health and education social service providers acknowledged and understood the key roles they played in making services accessible to their respective communities and oftentimes referenced their longstanding commitment and role in being a social service provider. For example, they described how they cultivated relationships with public and governmental organizations in their community and how they utilized relationships at times to broker favors for the populations they served. While most social service providers utilized their capital to support historically excluded populations, service providers at times revealed their political beliefs and/or understood that providing care was not an apolitical process. Accordingly, this means service providers can provide support, to a certain extent, at their own discretion. Their decision to provide support is informed by their personally held beliefs, values, and who they deem to be deserving of such help. For example, statements from interviewees, such as “We take care of our own” and “We know everyone,” were illustrative of their close-knit communities and an internal network that is assumed to be accessible by anyone in the community. However, the sociopolitical nature of insider/outsider dynamics and the ways service providers rely on support from faith-based communities, access to care can be determined by the social capital an individual, group, or population espouses, in addition to whether they possess membership in certain community groups. Below is an excerpt from an educator and health service provider on how he would navigate potential challenges when serving immigrant communities:

“One of the first things I would do is call our county health department over there, which we have developed over 12 years, a very close relationship, and just like this situation here, they will give me some advice…so then the administrator will take it to their PTA or PTO to try to get some help…well, let me say we don’t turn anybody over to ICE. We do not send anything to them. We’re going to deal with the child and the family and so what we’ll do is work through translators and so forth, we do have a—we have a person who works part-time here in our offices that worked for the county many years and has many connections in the county. They will help that father try to find a job, if needed, find somewhere to live, which is a problem in our county is housing, but they will try to find them a place to live temporarily so that that parent can actually start making some money, and then we’ll monitor them to—you know, they’ll do what they need to do as far as immunizations for the child, making sure they’re in school all the time and so forth. We’ll try to help them out as much as we can, if somebody else turns them in, we can’t help that, but we will not let—We will not let—We will not let the federal government on our campuses to pick people up as their getting their child or dropping them off and that sort of thing… I had to chase them [ICE] off one of our primary’s campuses last year.” (Coordinated School Health Director in rural county)

Additionally, changing populations across the state present challenges to families accessing services who oftentimes rely on service providers to assist them in navigating administrative requirements [9,36,37]. Population changes have also resulted in increased service needs for certain families; these changes include short- and long-term consequences of drug crises, the increasing number of grandparents and great-grandparents who serve as primary caretakers of their children, and a growing immigrant-origin population, particularly in predominantly White communities. Service providers in the study shared examples of complex situations they had to navigate, often because of the changing population in their community, that required them to provide additional support such as financial assistance or overcoming language barriers.

A service provider from a federally qualified health center in an urban county shared the following:

“We are able to do so little in those [high-risk] circumstances because on that, on top of that, it’s not uncommon for the dad to be suicidal or there’s someone in the home that is abusing alcohol or dad—and we’ve had this happen—dad is HIV-positive, and we find out the baby is HIV-positive. The mom is not there, so we don’t know. But we’ve had—now what do you do? And then they don’t have a place to stay. So, I’m going to just add that more to you because this is—this is our every day. We literally have—we’ve had—in our clinic 2 weeks in a row where the mother—was the father there? The father was okay. The mother was HIV-positive, and at least two out of the three kids were HIV-positive. And they didn’t even know. And we are like—and you know, they don’t speak English, so I’m just trying to see—this is our normal. That’s our every day.”

### 3.3. Salience of Place: Policies That Inhibit Access to Resources and Support 

Health and education service providers understand that policies and systems can significantly influence access to resources for families, particularly those facing additional barriers (e.g., income status or degree of “belonging” within a community). Interviewees described both having insufficient monetary funds to meet the growing needs of the populations they served and working to support marginalized populations that did not have access to resources or economic structures needed to break through various types of poverty cycles due to stringent programs and policies. Interviewees referenced several state- and federal-level policies in connection with barriers that service providers and families navigate across a variety of care settings and county types. In the following illustrative example, an interviewee describes how state-level policies around resource allocation had significantly impacted their ability to meet the needs of children and families:

“But we have a high rate of suicide and mental illness in the region, and I feel like that money should be allocated to areas that are in most need. But what I’m seeing a lot of times is, “Oh, we’re going to give it to the bigger places,” and what you have there is places that have more money, they have more resources, and then of course your impoverished areas, your small rural areas where nobody wants to come, we can’t even afford to hire anybody at this time because the money has been given to bigger places…. We need to establish funding that is more reoccurring to the district, and every district, every district on that. Last year, I was able to secure in-kind and grant funding for our district, and that is a huge help to us, especially when, you know, you’re in a really small district, and we don’t get a lot of funding anyway, especially when it’s based on [the state education funding formula]. They’re just not going to give it to us. And so, our kids—our kids do without. And probably I would think our kids have more of a need than, you know, some of the bigger schools, you know, get [funding]. You know, I know they have needs, but I doubt that their poverty rate and their mental health issues and their opioid issues here, it’s just not the same as it is here. I mean, we are in a crisis here.” (Coordinated school health director in a rural, economically distressed county)

Additional policies and systems that contributed to economic inequality and poverty among families with whom the interviewed service providers worked included: non-expanded Medicaid, non-livable minimum wage, increasing cuts to federal and state programs meant to support low-resource households (i.e., social safety net programs such as Supplemental Nutritional Assistance Program, Women Infants and Children, etc.), and decreasing or stagnant investment in health and education programs. These economic disparities were further exacerbated in rural counties and between rural counties due to non-comprehensive measures of poverty, non-equitable investments in employment opportunities across counties, and lack of affordable or physically accessible childcare options depending on where one lives. Interviewees discussed several examples of how these measurements of poverty and other county metrics used by the state disadvantaged their ability to be prioritized for additional resources due to low population density, among other factors. Such economic-based dynamics are especially detrimental to low-income and working poor families.

### 3.4. Intergenerational Experiences of Adversity within Communities

The majority of interviewees spoke in depth about the interwoven place-based factors that created high-risk conditions, as well as the intergenerational nature of ACEs and forms of adversity experienced by a high proportion of families within a community. In their descriptions, service providers often explained how environmental factors influenced family-level factors, family structure, and, subsequently, a child’s risk of ACEs. What follows are two illustrative quotes from service providers who link factors that shape high-risk conditions with their home life and respective ACEs linked to high-risk conditions. To provide additional context for the first excerpt, service providers from this particular drug coalition in a rural, economically distressed county described how high-poverty rates in their county had been the status quo since the 1970s due to the shutdown of the coal mining industry in their geographic area. For many generations, the majority of people living in their area did not have access to many full-time employment opportunities, jobs with adequate salaries, or the ability to develop employment skills. High-risk conditions for individuals, families, and their communities worsened due to an exponential number of pill mills that contributed to the opioid crisis before the government recognized the crisis. The service provider then proceeded to link these factors with various forms of adverse childhood experiences:

“There is an actual poverty rate, a 27.7% … but [what] that is saying [is accepting poverty rates]—which makes me so angry because we have said to these students, to this group of youth, “Hey, what are we going to do?” That it is okay. And it’s not. I mean, now try to tell those kids that they have more worth and value than that, you know? They’re living with drug-infested homes. They’re living with all of the problems. I mean, it’s not just mothers and fathers. It’s their grandmothers and grandfathers that are doing this. I had a young man tell me the other day that, you know, he was sitting at my feet, and he said—because he calls me grandma—he said, “I have watched my grandma take [motions injecting arm]—you know, tie off and shoot up in front of me and then she would pass out.” And he said that was so scary. And he said what was even scarier, when [he] had to spend the night and all the roaches in the house. You know, this is the reality of what these kids are really living with. And the principal at [the local high school] told me at the very beginning of this, she said, “Our students can’t come in here and worry about a chemistry test or, you know, what’s going on in high school when they’re more concerned about am I going to have food? When I get out at night, who’s going to pick me up from school? Will I be allowed to ride that bus? You know, are the things going to be taken care of for me?” And so, they have no worth and value, so they step right into the paths of their parents. They’re doing—making the same mistakes. This is generational mistakes in this community.” (Director of a community anti-drug coalition in a rural, economically distressed county)

The above illustrative quote references intergenerational drug abuse in the home accompanied by uninhabitable living conditions that present immediate health risks for children and families. The excerpt also references experienced food insecurity, transit insecurity, and an inability to rely on basic needs being met among children living in the community. According to service providers, the identified ACEs are experienced amid the consequences of social, political, and economic contexts that shape the salience of place. To showcase how the salience of place impacts intergenerational family histories, we present the following excerpt from a staff member who was responsible for working with youth within a drug coalition in a rural, economically distressed county:

“I had a young man who was brave enough to come down and I had all this [ACEs] logic model and all of my curriculum all set up just so perfectly, and he came down, he said, “I grasp the concept of what you’re trying to do here, and it’s good,” he said, “but you’re missing the mark.” And he began to really tell me, “You know, when you live in domestic violence, when you live in abuse—an abused home, and you’re—you become a bully, and you—you know, that deals with your mental health. That starts, you know, all your mental health issues that are going on during this, you turn to drugs and alcohol. That’s how we self-medicate.” … He took the black pen and really started writing, “This leads to homelessness.” You know, he’s a foster child. He came to [this] county because he was in foster care. This is—it just makes so much sense. If you can help them to understand these issues, what led us there … it helps you understand that we don’t have to go there…. Living in these issues, when you live like that, it becomes your comfort zone, even if you don’t like it. You become comfortable in this crazy, you know, wacky environment.” (Staff member of a community-anti-drug coalition in a rural, economically distressed county)

To provide additional context for the above quote, the staff person had described how this child had experienced additional challenges as foster youth having moved from another county without a “close to kin” foster parent. In this case, the child was not just a foster youth, but a child who resided in a physically isolated geographic area, without familial mentorship to meet basic child development needs, who had a debilitating self-esteem as a resident of an economically distressed and rural county. The various identities that are referenced by the child are as follows: unhoused (homeless), victim of domestic violence, compromised mental health status, substance use disorder, intergenerational unhoused status. This quote highlights how high-risk conditions permeate family histories and impact generations. 

### 3.5. Recognizing Additional Subpopulations and Identities

**Figure 1:**
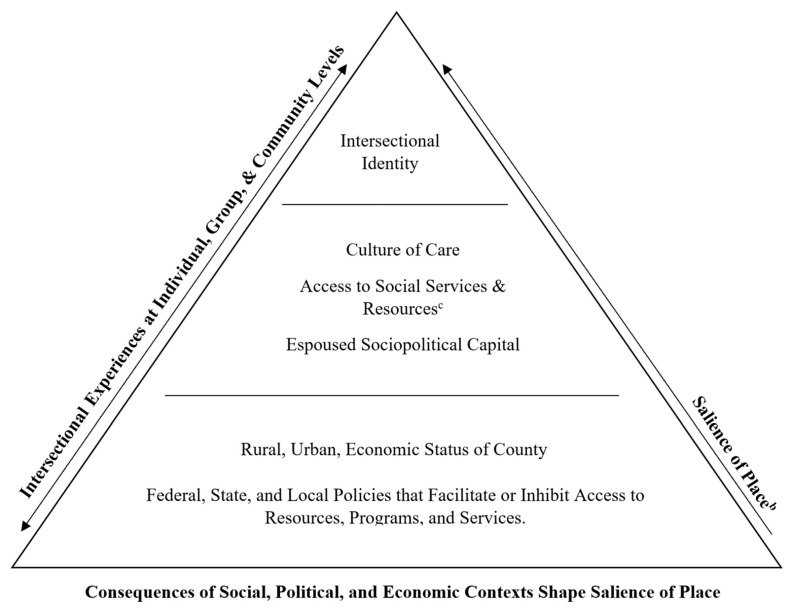
The *Intersectional Nature of ACEs Framework* ^a^. Note. ^a^ Subpopulations experience high-risk conditions as a result of social, political, and economic contexts at individual, group, and community levels due to systemic inequality. Subpopulations identified in our interview data did not comprise an exhaustive list of place-based determinants. To see the comprehensive list of place-based determinants derived from this study which inform the relationship between the salience of place and intersectional experiences, see the Appendix A. ^b^ Place-based contexts can determine high-risk conditions and are constructed by the physical nature of place, as well as governing policies, systems, and processes that determine access and availability of resources, programs, and services. However, access to resources, programs, and services is additionally materialized by the culture of care and how individuals, groups, and communities are recognized. Community characteristics can include the physical location of resources, transportation access, and changes in population and place-based crises that compound high-risk conditions (e.g., opioid crisis). ^c^ Access to social services and resources mitigates factors that construct high-risk conditions which in turn lessen ACEs.

Guided by process-centered intersectionality, we identified subpopulations (see Appendix A) not commonly accounted for in research studies. Service providers identified these subpopulations according to their social and economic standing, place of origin, geographic location, family history, and experienced high-risk conditions. Our data analysis excavated 119 identities, either referenced directly by service providers or derived from the qualitative data analysis. While some identities are broadly recognized (i.e., race, gender, age, family structure), we compiled a list of additional identities that may contribute to one’s likelihood of exposure to ACEs or one’s likelihood of experiencing barriers when accessing services. From that point of reference, we developed the *Intersectional Nature of ACEs Framework*, illustrated in Figure 1. 

*The Intersectional Nature of ACEs Framework* illustrates that ACEs compounded by high-risk conditions are first and foremost undergirded by the consequences of social, political, and economic contexts which in turn shape the salience of place. The salience of place is not experienced during a particular moment; rather, the relationship between policies at federal, state, and local levels and the demographic and economic composition of place determine access to resources. This in turn shapes a dynamic culture of care that determines experienced access to resources among historically excluded populations and subpopulations per their espoused sociopolitical capital. Ultimately, intersectional identities across ecological levels are a result of the dynamism of high-risk conditions which can be traced back to systemic inequality. To illustrate the relationship and centrality of the consequences of political, social, and economic contexts, the preceding co-constructs the salience of place and intersectional experiences across ecological levels. 

The quotations and accompanying descriptions in Table 1 exemplify the intersection among ACEs, salience of place, and high-risk environments, informed by the *Intersectional Nature of ACEs Framework*. This framework builds upon previous ACEs frameworks, highlighting underlying and often upstream factors that may connect ACEs within the life of a child, a family unit, or a community. Operationalizing an intersectional lens in the study of ACEs allows for a non-cumulative measure of adversity that is not only affected by how many ACEs one experiences, but also by intersectional identities one can possess, thereby examining compounding effects of ACEs. Table 1 includes examples of ACEs experienced by children within various place-based contexts; these contexts have varying risk levels of ACEs and are also affected by broader policies and systems that affect marginality. Thus, the *Intersectional Nature of ACEs Framework* emphasizes the multiple levels of risk factors that affect exposure and access to resources, focusing on high-risk environments and upstream factors to expand upon previous approaches that primarily emphasize family and individual-level factors. 

Collectively, these findings illustrate a more complex understanding of ACEs and how service providers experience the context of their community and/or their capacity to support these families. Identifying these high-risk conditions highlights the need to respond to ACEs not only on an individual level, but also on family, community, and state levels. 

## 4. Discussion

Inequitable environments are powerful forces that impose additional identities upon historically excluded populations. Children, families, and communities living in high-risk environments who are experiencing ACEs may have difficulty accessing resources due to the intersecting identities they possess. In this study, the high-risk conditions described by service providers across Tennessee illuminate the environmental factors contributing to co-occurring ACEs and challenge scholars and practitioners to reconceptualize the way programs and policies support these families. While original ACEs research focused on adverse childhood experiences individually as events to examine using a cumulative measure, we encourage program administrators to focus on high-risk environments where multiple ACEs may be connected by underlying mechanisms; we also recommend that public administration officials focus on policy solutions that allow families to break out of generational poverty that may contribute to the risk of experiencing ACEs. Inability to access services while living in poverty may specifically contribute to neglect, maltreatment, and foster care. The opioid crisis in Tennessee, for example, and subsequent high-risk environments may facilitate the co-occurring ACEs of substance abuse in the household, mental illness in the household, various types of abuse, foster care, death of a family member, and neglect. Evidently, health and education service providers must consider the basic needs (e.g., adequate food and housing) of historically excluded populations with their specific services. Program administrators should prioritize funding programs that allow providers to care for families and children comprehensively, with additional training on responding to a high-risk situation holistically instead of only assessing a child’s well-being with a traditional ACEs screening tool.

Considering the *Intersectional Nature of ACEs Framework* that emerged from this study, we emphasize the importance of developing health and education resources and services with practices that maximize safety nets. First, when possible, service providers should incorporate cradle-to-grave (lifelong) support and programming. As evidenced in this study, the challenges that historically excluded populations must navigate are many, and it will be impossible for them to navigate complex systems without major reform of current processes. To offset compounding effects from high-risk environments, service providers should redevelop current programs and services to meet the needs of diverse populations, regardless of which stage-in-life services are accessed. This safety-net approach will meet historically marginalized populations within their experienced reality, as well as mitigate the consequences of inequitable environments that ultimately rob people of their agency, human dignity, and ability to realize their full selves. Second, given that ACEs may be rooted in intergenerational high-risk conditions, service providers should assume multigenerational support as a form of safety net. This approach will enable services and resources to be developed for different-aged populations with the understanding that the objective is to prevent younger generations from inheriting high-risk conditions to break out of high-risk conditions. In the interviews, the multigenerational approach was described as vital by service providers as well as ACEs researchers and providers who had developed programs serving multiple family members together in high-risk situations, such as addiction and parent history of ACEs [38,39]. 

Situated at the frontlines, service providers can mitigate ACEs and espouse tremendous social capital; they have the agency to either discriminate or use their knowledge to help historically marginalized populations navigate complex social service systems. We strongly advise a higher level focus on improving policies and systems that help families break out of generational poverty and intergenerational cycles of ACEs—mobilizing for under-insured and uninsured populations, exposing the depths of poverty in their communities, educating policymakers, working across organizations to increase protections for the working poor, etc.—so that historically excluded populations can think beyond their immediate survival and work toward realizing intergenerational mobility. 

Previous studies have expanded upon the 10 traditional ACEs but have not necessarily contextualized high-risk, inequitable environments and multidimensional elements of place that intersect with ACEs. With the *Intersectional Nature of ACEs Framework* in mind, we present the term *repression-ACEs* to differentiate between ACEs that are the consequences of social inequities, such as neighborhood violence and racism, and ACEs that are inflicted directly by a person. Repression-ACEs signals the ways in which ACEs are constructed by higher-risk environments that are the consequences of social, political, and economic contexts that shape the salience of place. We hope this term will differentiate the power of imposed-upon environments and the collective responsibility to disrupt harmful policies and systems.

## 5. Conclusions

Espousing a critical intersectional approach and utilizing in-depth interviews to understand the ACEs phenomenon, this study’s results make a significant and interdisciplinary contribution to the ACEs literature and deepens understanding of the social problems service providers navigate. When mitigating ACEs through prevention and support, scholars and practitioners need to consider the precarious place in society children inhabit and how policies and programs have the potential to worsen high-risk conditions. If scholars, policymakers, and practitioners provide high-risk communities with sufficient resources and support, the collective efforts can hopefully prevent children from experiencing the very worst consequences of childhood abuse, neglect, and household dysfunction.

## Figures and Tables

**Table 1:** High-Risk Conditions, Environments, and Their Relationship to ACEs.

Consequences of Social, Political, and Economic Contexts	Environment	High-Risk Conditions (Local)	High-Risk Conditions (Familial)	ACE(s)	Illustrative Quote
Unemployment loss for decades	Highest unemployment rate in the state of Tennessee	Rural, economically distressed county with prevalent food, housing, and transit insecurity	Unemployed guardians	Neglect	“Well, we were kind of used to recession and poverty because it started back in the ‘70s, okay? So, we was kind of used to that, but we didn’t really know how to evaluate and identify because we had to get ourselves trained, and that’s why it was so good when the coalition concept come in and really showed us and trained us on how to evaluate our population, and then everybody went through a decline economically for a long time. We went from where you could not get a job…. The unemployment rate at one time was up to 26%. We had the highest unemployment rate in the state. Five years in a row. Five. In the mid-2000s. Because there wasn’t a lot of opportunity here, okay? The factories wasn’t—weren’t—we’re a manufacturer type population. We’re not a highly skilled labor population, okay? So, excuse me, we went through that, then we seen the drug epidemic starting, and really, I mean, it started as a means of sustainability, people selling their medications to pay their electric bill, and a self-coping mechanism. People were using it because they were depressed. Self-medicate. So that population and the opioid population, along with the country, just became an epidemic here.”
Economic recession	Intergenerational poverty		Experienced poverty in home		
Opioid crisis	Intergenerational drug use	Insufficient resources and services among organizations to address addiction experienced among individuals, families, and communities	Experienced drug use disorder in home	Substance use in household	
Measures of poverty that determine government housing programs for rural counties	High unemployment rate; intergenerational absence of workforce development	Insufficient resources and services among organizations to address prevalent food and housing insecurity	Public housing resident; government housing does not include childcare center	Sexual Abuse	We have seen some increased instances in the last couple of years at our properties [government public housing] of some instances of abuse of children, and the children weren’t school aged children. They were younger. So I had talked with—let’s maybe partner and do a training for the parents to come, and a lot of the time participation when we’d try to have types of trainings and things at the housing authority participation is generally low, and so that—We see that sometimes [childcare] as a barrier to things that we want to try to plan, because you know, we can plan all kinds of things, but we’ve tried to partner with the health department on smoking—you know, just different things … health literacy … A lot of it [child sexual abuse] has to do with substance use disorder in our community and how prevalent it is and these children get taken away in those situations a ton. That’s mainly what I would say the majority of the children come from…neglect, abuse … I would say a lot of it stems from the substance use disorder problem that we have in our community and that’s why we have a lot of those referrals … there is a lot of sexual abuse with substance abuse. A lot of the times, talking about what we were a while ago, parents trying to work and people watching the kids and they just drop them off with whoever [facilitates child sexual abuse].”
Economic development policies that permit disparate socioeconomic status	Most available employment opportunities are minimum wage jobs; earned wages do not meet basic needs of families	Absence of safe, affordable childcare for the working poor	Guardian and/or parent(s) need to earn a certain income amount to remain in government housing program thus ability to maintain housing security is linked to childcare	Neglect	
Opioid crisis	Intergenerational drug use	Insufficient resources and services among organizations to address addiction experienced among individuals, families, and communities	Childcare provider has substance use disorder	Substance abuse by caregiver	

## Data Availability

Interview data used in the study cannot be publicly shared.

## Appendix A. Subpopulations at Individual, Group, and Population Identities

Sub-populations are not commonly accounted for in research studies. These sub-populations were identified by service providers according to their social and economic standing, place of origin, geographic location, family history, and experienced high-risk conditions. The expanded-upon populations in this list present additional information about the experience that was referenced by health and social service providers. Higher-risk environments construct circumstances that may contribute to higher-risk conditions and worsen effects of ACEs. Each of these identities were referenced directly or indirectly from our interviews with 81 service providers.

### Appendix A.1. Individual Identities Attributed to a Child, Family Member, or Individual Living in Their Household Due to the Consequences of Social, Political and Economic Histories and the Salience of Place

1. Race
2. Gender
3. Sexual orientation
4. Religious orientation
5. Socioeconomic status
  Income
  Access to reliable transportation
6. Residency status
  U.S. citizen
  Undocumented U.S. citizen (i.e., cannot afford birth certificate)
  Permanent resident
  Refugee
  Temporary workplace visa (i.e., H2A visa)
  Undocumented person
7. Immigrant country of origin
8. Place of origin
9. Place-based identity (Appalachian, Southern, etc.)
10. New place-based identity (relocates to live with grandparent/great grandparent due to home life instability)
11. Native to Tennessee
12. Drug disorder
13. Drug disorder family history
14. Supplemental Nutrition Assistance Program (SNAP) recipient
15. Supplemental Nutrition Assistance Program (SNAP) recipient family history
16. Special Supplemental Nutrition Program for Women, Infants, and Children (WIC) recipient
17. Special Supplemental Nutrition Program for Women, Infants, and Children (WIC) recipient family history
18. Temporary Assistance for Needy Families (TANF) recipient
19. Temporary Assistance for Needy Families (TANF) family history
20. Incarcerated status
21. Incarcerated family history
22. Personal criminal record
23. Active probation status
24. Newly released from prison, rehabilitation, or halfway house
25. Medicated Assisted Therapy recipient
26. Long-term Medicated Assisted Therapy participant becomes dependent on therapy
27. Church affiliation
28. Health status
  Acute disease status
  Chronic disease status (asthma, diabetes, ADD/ADHD)
  Mental health status
  Learning disabilities
29. Education
  Formal education
  Literacy level
  Digital literacy level
30. Language
  Native English speaker
  Non-English speaker
  English as a second language
  English as a third language, Spanish as a second language (Indigenous language is native language)
31. Employment
  Employed
  Working Poor
  Underemployed
  Unemployed
  Exploitative work conditions
  Underserved (i.e., no access to healthcare benefits)
  Non-highly skilled
  Non-highly skilled, manual intensive labor
  Essential
32. Housing
  Housed
  Housed without basic necessities (e.g., running water, electricity)
  - Housing insecure
  - Overcrowding
  Transitional resident (degree of homelessness)
  Unhoused (homeless)
33. Health Insurance
  Insured
  Uninsured
  Unknown loss of insurance
  Experienced insurance-gap

### Appendix A.2. Family (Group) Identities That Are Part of Communities Whose Circumstances May Establish Higher-Risk Conditions for Children and Compound ACEs

1. Mixed status families
2. English as a second language
3. Non-English speakers
4. Kin Foster Parents
5. Close to kin, lives in same county
6. Non-kin Foster Parents
7. Foster youth
8. Guardian grandparents and great grandparents
9. Temporary kin guardians
10. Special needs guardians
11. Residents of immigration raid county and perceived of being Latinx/e origin and/or undocumented
12. Residents of immigration raid whose fear of being profiled prohibits their ability to drive, travel, and seek health and education support services
13. Residents of distressed county
14. Residents of rural county
15. Residents of a high-risk geographic area (proximity to pill mill, cartel route, Appalachia, mountainous, and/or physical divide that perpetuates socioeconomic stratification, etc.)
16. Residents who do not have access to a hospital or urgent care (healthcare desert)
17. Women who live in healthcare deserts and do not have access to women’s healthcare
18. Residents without emergency shelter services
19. Residents who do not have a church affiliation and/or faith-based support system and thus have lesser access to resources and services
20. Residents who experience a faith-based divide by race, sexual orientation, socioeconomic status, etc.
21. Residents who are not in close proximity to their faith-based community and/or leadership
22. Residents of a geographic area with higher concentrations of an elderly population and children population, limited employment opportunities, and shrinking middle class
23. Residents of a geographic area with no public transportation
24. Residents of a geographic area with no access to Lyft, Uber, or taxi services
25. Residents who live in a community with high service provider turnover
26. Residents without WIFI infrastructure/access
27. Residents who live in homes that are not sufficiently insulated and/or are under resourced housing communities without basic services (e.g., trailer parks)
28. Residents that live in homes that are overcrowded
29. Residents that are part of public housing or reduced housing program
30. Service providers who experience secondary trauma, burnout, and/or are not supported due to limited organizational resources and personnel
31. Mandated reporters
32. Halfway house residents
33. Incarcerated population
34. Incarcerated population without access to Medicated Assisted Therapy
35. Family members with warrants
36. Family members who take care of indigent family members
37. Family members with special food needs (special needs baby formula, food allergies, etc.) who experience additional WIC/SNAP limitations
38. Family reputation in tight knit community
39. Pregnant mothers with drug disorder
40. New mothers with neonatal opioid withdrawal syndrome (NOWS) baby
41. Non-involved parents/caretakers as it pertains to school
42. Guardians who only have gas money to travel to-and-from work
43. Elderly on fixed income or low-income status
44. Elderly who experience a technological divide that constrains access to health and public services
45. Elderly that cannot drive
46. People who have been dropped from receiving educational and/or health services due to grant start-stop grant cycle
47. People who experience stigma for seeking mental health services
48. People on waiting lists for emergency and/or public services (e.g., public housing, Medicated Assisted Therapy bed, etc.)
49. People who purposely get arrested to access healthcare and dental needs
50. Victims of domestic violence
51. Victims of natural disasters
52. Populations that are more likely to be prescribed opioids
53. Populations with predisposition to drugs (as described by service providers)
54. Uninsured population when the child turns 18 years of age and no longer qualify for TennCare until they are 19 years of age

### Appendix A.3. Group (Child) Identities Whose Familial Circumstances Linked to an Identity May Develop Higher-Risk Conditions That Worsen ACEs

1. Frequent flyers (children that see the nurse on an ongoing basis because they have underlined health issues and do not have a primary care physician)
2. Children separated from parent(s) due to immigration status
3. Children who experienced temporary separation due to immigration status
4. Children who are chronically absent from school for health-related reasons
5. Children who reside in a physically isolated geographic area
6. Children who are caretakers of self, siblings, and/or their parents
7. Children who work and miss school to help support their parents, grandparents, etc.
8. Children with no house phone and/or changing phone number
9. Children who rely on free and reduced lunch for regular meals
10. Children who experience food insecurity over the weekend and/or end of the month when SNAP benefits are no longer available
11. Children who are transitional (coming back from a facility)
12. Different-aged children and their experience and exposure to opioids
13. Children living in a house of a former partner or spouse (unrelated) to the guardian
14. Children living in close proximity to a registered sex offender
15. Children who have bed bugs, lice, or other contagious pests in home
16. Children who self-harm
17. Children with disabilities
18. Children with eating disorders
19. Children who have suicide ideation
20. Children who are on a waiting list to access services (i.e., Head Start)
21. Custodial children
22. Children without familial mentorship and support to meet basic child development needs
23. Children with debilitating self-esteem
24. Children who do not have access to transportation beyond relying on the school bus
25. Children who survive a familial or peer suicide
26. Children who survive familial or peer drug overdose
27. Children who were dropped from the healthcare system when seeking mental health services due to service provider employment retention
28. Children in the juvenile incarceration system
29. Children whose parent(s) are in rehabilitation
30. Children who experience drug use disorders in home
31. Unhoused male children over 12 years of age (cannot stay with mother in homeless shelter facility)
32. Children who suffer from neonatal opioid withdrawal syndrome
